# Sebaceous gland reprogramming with a single gene, *PPARG*, and small molecules

**DOI:** 10.1038/s41392-023-01531-3

**Published:** 2023-08-04

**Authors:** Yiqiong Liu, Shuaifei Ji, Huanhuan Gao, Huating Chen, Jiangbing Xiang, Shaoyuan Cui, Christos C. Zouboulis, Aizhen Cai, Xiaobing Fu, Xiaoyan Sun

**Affiliations:** 1 Research Center for Tissue Repair and Regeneration affiliated to the Medical Innovation Research Department and 4th Medical Center, PLA General Hospital and PLA Medical College; PLA Key Laboratory of Tissue Repair and Regenerative Medicine and Beijing Key Research Laboratory of Skin Injury, Repair, and Regeneration; Research Unit of Trauma Care, Tissue Repair and Regeneration, Chinese Academy of Medical Sciences, 2019RU051, 100048 Beijing, P. R. China; 2 Department of Nephrology, The First Medical Center, Chinese PLA General Hospital, State Key Laboratory of Kidney Diseases, 100048 Beijing, China; 3 Departments of Dermatology, Venereology, Allergology, and Immunology, Staedtisches Klinikum Dessau, Brandenburg Medical School Theodor Fontane and Faculty of Health Sciences Brandenburg, Dessau, Germany; 4 Institute of General Surgery, Department of General Surgery, the 1st Medical Center, PLA General Hospital, 28 Fu Xing Road, 100853 Beijing, P. R. China

**Keywords:** Reprogramming, Skin stem cells

**Dear Editor**,

Sebaceous glands (SG) contribute to 90% of the skin surface lipid production and play critical roles in regulating proper skin functions including epidermal barrier maintenance, dermal immune responses, and global body anti-microbial activities.^1^ Reduced sebaceous lipid has been implicated in diverse skin diseases, including scarring alopecia, inflammation, chronic itch, as well as premature aging of the skin.^2^ Especially, scar repair after deep burns or large-scale skin defect often leads to irreversible loss of appendages. Due to lacking SG, the regenerated tissues cannot fully recapitulate the structural and functional integrity of normal skin. Therefore, generation of expandable SG cells with developmental and lipid-producing potentials represents a promising approach for treating diseased, damaged, or aged skin with better quality of life. Recently, direct cellular reprogramming has been widely used for obtaining difficult-to-maintain human cells by cell type-specific transcription factors (TFs). Small molecules are also used to promote TF-mediated cellular reprogramming and shorten the process of generating safer customized cells for transplantation. However, the conversion of human somatic cells into SG cells has not been investigated so far. Here, we described a step-wise reprogramming strategy that permits the generation of expandable and functionally competent human SG cells.

Human epidermal keratinocytes (HEK) isolated from adult foreskin showed no contamination of SG cells as assayed by qRT-PCR and immunofluorescence (Supplementary Fig. S1a, b). Comparative transcriptome analysis between HEK and primary SG cells (pSC) revealed that the upregulated differentially expressed genes (DEG) were significantly involved in several biological processes relevant to adipogenesis and lipid metabolism (Supplementary Fig. S2a), in which PPAR signaling pathway was enriched by KEGG analysis (Fig. 1a). Accordingly, PPARγ was selected as a potential SG fate-determining factor owing to its unique role in SG specification and patterning.^3,4^ To induce SG conversion, HEK stably overexpressing PPARγ (HEK-PPARγ) were generated using lentiviral *pLV-CMV-PPARγ-P2A-EGFP* vector (Supplementary Fig. S2b, c). KEGG analysis showed that the upregulated DEG were mainly enriched in lipid-related pathways, and PPAR signaling pathway was activated after PPARγ overexpression (Supplementary Fig. S2d). Then, we switched HEK-PPARγ to the SG induction medium (SGIM) containing SG organogenesis molecules EGF, FGF7 and FGF10. HEK-PPARγ cultured in SGIM were thereafter referred as HPSM. After 3 days of exposure, HPSM with SG cell-like morphology emerged (Supplementary Fig. 2e). qRT-PCR showed that several key SG genes including *AR*, *GATA6*, *CK7*, as well as SG differentiation markers *MUC1* and *PLIN2*, were markedly upregulated on day 6 (Fig. 1b). Further immunofluorescence demonstrated the percentage of HPSM expressing MUC1, CK7 and FASN was 29.2 ± 3.2%, 37.2 ± 2.7%, and 52.9 ± 6.6%, respectively (Supplementary Fig. S2f, g).

**Fig. 1:**
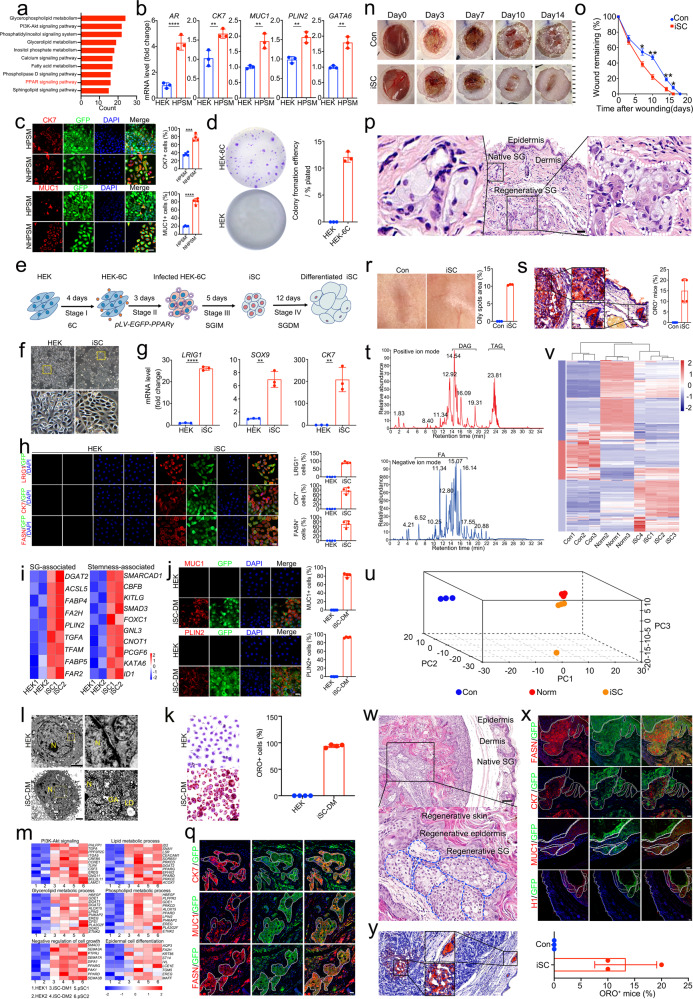
Sebaceous gland reprogramming with a single gene, *PPARG*, and small molecules. **a** KEGG analysis of upregulated lipid-related genes in pSC compared with HEK. pSC, primary SG cells. **b** qRT-PCR analysis of transcriptional expression of *AR, CK7, MUC1, PLIN2*, and *GATA6* in HEK and HPSM. Data are expressed as mean ± S.D.; *n* = 3. **p < 0.01; ****p < 0.0001. HPSM, HEK stably overexpressing PPARγ and cultured in SGIM. **c** Representative immunofluorescence of CK7 and MUC1 in HPSM and NHPSM (left) and quantitative analysis of CK7^+^ and MUC1^+^ cells in HPSM and NHPSM (right). NHPSM, neonatal HEK stably overexpressing PPARγ and cultured in SGIM; Scale bar = 50 μm. Quantification was done with four randomly selected individual microscopy fields. **d** Representative images of colony forming unit (CFU) assays in HEK and HEK-6C (left) and quantification of their colony forming efficiency (right). *n* = 3. HEK-6C, HEK treated with chemicals 616452 (6) and CHIR99021 (C). **e** Schematic view of the iSC reprogramming diagram. HEK treated with 6C (HEK-6C) for 4 days were transduced with PPARγ and plated in HEK medium. Then, the infected HEK-6C cells were transferred into SGIM and cultured for indicated days. iSC, induced sebaceous gland cells. SGIM, sebaceous gland induction medium; SGDM, also called DM, sebaceous gland differentiation medium. **f** Phase contrast images showing the obvious morphological changes of iSC compared to HEK. Scale bar = 100 μm. Insets, higher magnification of the boxed areas. **g** qRT-PCR analysis of transcriptional expression of *CK7*, *LRIG1*, and *SOX9* in HEK and iSC. Data are presented as mean ± S.D.; *n* = 3. **p < 0.01; ****p < 0.0001. **h** Representative immunofluorescence of LRIG1, CK7, and FASN in HEK and iSC (left) and quantitative analysis of LRIG1^+^, CK7^+^, and FASN^+^ cells in HEK and iSC (right). Scale bar = 25 μm. Quantification was done with four randomly selected individual microscopy fields. **i** Heatmap of stemness- and SG- associated genes in iSC compared to HEK (adjust *P* value < 0.05). **j** Representative immunofluorescence of MUC1 and PLIN2 in HEK and iSC-DM (left) and quantitative analysis of MUC1^+^ and PLIN2^+^ cells in HEK and iSC-DM (right). Scale bar = 25 μm. Quantification was done with four randomly selected individual microscopy fields. iSC-DM, iSC cultured in sebaceous gland differentiation medium (DM). **k** Representative images of Oil red O (ORO) staining in HEK and iSC-DM (left), and quantitative analysis of ORO staining positive cells in HEK and iSC-DM (right). Scale bar = 25 μm. Quantification was done with four randomly selected individual microscopy fields; **l** Ultrastructure analysis of HEK and iSC-DM. Scale bars = 2000 nm. Insets, higher magnification of the boxed areas. N, Nuclear; GA, Golgi apparatus; LD, lipid droplet. **m** Heatmap depicting the representative KEGG pathway and GO terms of gene expression among iSC-DM, pSC and HEK (adjust *P* value < 0.05). **n** Representative macroscopic images of wound healing at indicated days in control and iSC-treated mice. **o**. Quantification of remaining wound areas on day 0, 3, 7, 10, 14, 16, and 18 in the control and iSC-treated mice relative to original area, respectively (*n* = 40, per group). *p < 0.05; **p < 0.01. **p** Representative H&E staining in iSC-treated mice at day 71. Scale bars = 50 μm. Insets, higher magnification of the boxed areas. **q** Immunofluorescence of SG marker expression in de novo SG structures on day 71 after iSC-treatment. GFP^+^ cells were observed in the SG-like structures, and the regenerated SG expressed SG-specific markers including CK7, FASN, and MUC1. Scale bar = 25 μm. **r** Representative macroscopic images of the regenerated skin in control and iSC-treated group (left) on day 71. Quantitative analysis of (right) skin greasiness from 3 independent experiments. **s** Representative ORO staining of the regenerated skin in iSC-treated mice (left) on day 71. Scale bars = 50 μm. Insets, higher magnification of the boxed areas; ORO staining assessment (right) of de novo SG showing, after 71 days treatment with iSC, 15.00 ± 2.887% of skin samples from the recipient mice (*n* = 40) were ORO staining positive. Data are represented as mean ± S.D. **t** Base peak chromatogram of skin samples from iSC-treated mice acquired in positive (up) and negative (down) ionization mode. DAG, diglycerides. TAG, triglyceride. FFA, free fatty acids. **u** PCA loadings plot showing three major directions identified among control, iSC-treated, and normal skin samples accounting for 92.6% of total variance. The main effect is associated with SG features where loadings are clustered closely between the iSC-treated and normal skin samples, but distinctly from control group. **v** Normalized intensities of lipid compounds identified in the control, iSC-treated, and normal skin samples. Ten columns are arranged by unsupervised hierarchical clustering, while the rows are arranged by results of *k*-means clustering. **w** Representative H&E staining of the regenerated skin in mice subcutaneously transplanted with iSC at day 112 post-operation. Emerging skin-like structures containing keratinized epidermis as well as underlying SG-like structures were seen in the iSC-transplanted mice. Dotted line represents the outer border of ectopically regenerated glandular architecture. Scale bars = 100 μm. Insets, higher magnification of the boxed areas. **x** Immunofluorescence of SG marker expression in the ectopically regenerated SG-like structures on day 112 post-transplantation. PPARγ-GFP^+^ cells were observed in the ectopically regenerated SG-like structures. Immunofluorescence of human-specific histone protein (H1) further confirmed human origin of the ectopically regenerated SG-like structures. Ectopically regenerated SG showed SG morphological features and expressed SG-specific markers including CK7, FASN, and MUC1. Dotted line represents the outer border of ectopically regenerated glandular architecture. Scale bars = 25 μm. **y** Representative ORO staining of SG regenerated ectopically on day 112 (left). ORO staining assessment (right) of the ectopically regenerated SG involved control and iSC-treated skin samples from 3 independent experiments. After 112 days treatment with iSC, 13.33 ± 3.33% of the recipient mice (*n* = 40) exhibited lipid-containing ectopic SG as compared with control group (*n* = 40). Scale bars = 50 μm. Insets, higher magnification of the boxed areas. Data are represented as mean ± S.D

To test the reliability of our induction strategy, neonatal HEK (NHEK) were also transduced with PPARγ (NHEK-PPARγ) (Supplementary Fig. S3a, b). Similarly, SG cell-like morphology appeared after 3 days of SGIM induction (Supplementary Fig. 3c). The increased expression of CK7 and MUC1 was confirmed by qRT-PCR and immunofluorescence (Supplementary Fig. 3d–f). Notably, the conversion efficiency of NHEK was higher than that of adult HEK, evidenced by immunofluorescence of CK7 (77.0 ± 4.3% vs. 36.4 ± 2.1%) and MUC1 (80.9 ± 3.9% vs. 19.9 ± 0.6%), suggesting that NHEK were more susceptible to cellular reprogramming than adult HEK (Fig. 1c). Moreover, qRT-PCR and immunofluorescence revealed that stemness-associated genes were highly expressed in NHEK (Supplementary Fig. S4a, b). CFU assay results showed that NHEK possessed a strong clonal growth capacity (Supplementary Fig. S4c, d). We thus hypothesized that additional factors that enhance stemness may boost the efficiency of SG reprogramming. To this end, two reported chemicals 616452 (6) and CHIR99021 (C) were added to improve SG conversion based on their known roles in enhancing stemness.^5,6^ Expectedly, qRT-PCR and immunofluorescence showed upregulated expression of stemness-associated genes in the 6C-treated HEK (Supplementary Fig. S5a, b). CFU assays further demonstrated that 6C pretreatment triggered an enhanced ability to form larger colonies (Fig. 1d).

Given these results, we designed the strategy to systemically reprogram HEK into induced SG cells (iSC) by combining 6C pretreatment, PPARγ overexpression, and SGIM culture (Fig. 1e). The induced cells grew rapidly and displayed typical tight aggregates with SG cell-like morphology at day 12 (Fig. 1f). Additionally, mRNA levels of *CK7* and SG progenitor markers *LRIG1* and *SOX9* were significantly upregulated in iSC (Fig. 1g). Immunofluorescence revealed that iSC expressed LRIG1, CK7, and FASN, demonstrating a gain of SG progenitor phenotype (Fig. 1h). Further genome heatmap showed that the iSC resembled their in vivo counterparts in terms of expression profiles including lipid synthesis genes, fatty acid binding protein genes, and lipid metabolic-associated transcription factors. TFs that modulate stem cell maintenance and signaling components that regulate progenitor proliferation were also enriched in iSC, suggesting the converted iSC exhibited molecular features of SG progenitors (Fig. 1i).

To characterize their functional properties in vitro, iSC were transferred into SG differentiation medium. After 10–14 days of differentiation, the iSC exhibited typical polygonal morphology (Supplementary Fig. 6a). Immunofluorescence analysis demonstrated that iSC cultured in SG differentiation medium exhibited dramatically increased percentage of MUC1^+^ cells (81.9 ± 2.4%) and PLIN2^+^ cells (92.6 ± 0.6%) (Fig. 1j). Ultrastructure analysis of the differentiated cells showed typical lipid-producing organelles including Golgi apparatus and lipid droplets (Fig. 1l). Remarkably, functional examination by ORO staining showed massive accumulation of lipid droplets in the differentiated iSC, suggesting that iSC can mimic native SG progenitors with lipid synthesis activity upon differentiation (Fig. 1k). By karyotype analysis, both iSC and iSC-derived differentiated sebocytes were stably expandable in vitro with normal karyotypes (Supplementary Fig. 6b). Additional gene expression profiling by RNA-seq showed that the differentiated iSC displayed expression patterns like primary SG cells in a set of genes involved in lipid metabolism, glycerolipid metabolism, and phospholipid metabolism (Fig. 1m).

To investigate their tissue reconstitution potential in vivo, the mixture of iSC and human fibroblasts or fibroblasts only (as control) were injected intradermally into a pilot full-thickness excisional wound. Compared to control group, a significant acceleration of wound closure was observed in the iSC transplantation mice (Fig. 1n, o). Moreover, well-organized de novo glandular structures with SG features were seen within the dermis of iSC-transplanted mice by 71 days (Fig. 1p). Tissue sections of the harvested wounds demonstrated that GFP-marked iSC progeny survived, and clusters of GFP^+^ cells expressing MUC1, FASN, and CK7 were observed extensively in the newly formed SG (Fig. 1q). Subsequent greasiness assessment of the re-epithelialized skin showed that iSC-treated wounds exhibited greasier than control wounds (Fig. 1r). ORO staining confirmed that, similar to native SG, the regenerated SG have lipid synthesis function, and approximately 15 ± 2.9% of the iSC-treated mice showed positive ORO staining (Fig. 1s). Lipid profiles characterization by high-performance liquid chromatography coupled with mass spectrometry revealed that a specific spectrum of lipids, including triacylglycerols, diacylglycerols, and free fatty acids, were enriched in the regenerated skin of iSC-treated mice (Fig. 1t). Further principal component analysis (PCA) and hierarchical clustering analysis showed that lipidomic profiles of the regenerated skin from iSC-treated wound were clustered closely with those from normal skin (Fig. 1u, v), indicating that iSC could facilitate functional SG regeneration.

To figure out whether the regenerative ability of iSC might be affected by foreign microenvironments, we next challenged the regenerative ability of iSC by transplanting them into subcutaneous muscle layer. After 112 days, ectopically regenerated skin-like structures were seen in the sub-fascia (Fig. 1w), which possessed keratinized multilayered epidermis and dermis containing SG-like structures with visible glandular domain (Fig. 1w). Immunofluorescence confirmed the expression of FASN, CK7, and MUC1 in the ectopically regenerated SG by iSC (Fig. 1x). Besides, human-specific histone protein H1 further confirmed the human origin of GFP^+^ SG glandular structures (Fig. 1x). Finally, ORO staining showed that the ectopically regenerated SG had lipid synthesis function, and approximately (13.33 ± 3.3%) of the mice were positive for ORO staining (Fig. 1y).

In conclusion, human SG cells with regenerative capacity can be generated by single factor-directed lineage reprogramming, highlighting potential therapeutic implications for personalized wound healing and delaying skin aging.

## Supplementary information

Supplementary Materials-

## Data Availability

Data that support the findings of this study are available from the corresponding author upon reasonable request.
