# Supplementary material for: Sebaceous gland reprogramming with a single gene, PPARG, and small molecules
Source: Signal Transduct Target Ther. 2023 Aug 4;8:286. doi: 10.1038/s41392-023-01531-3 (PMC10400560; doi:10.1038/s41392-023-01531-3)
Supplement: Supplementary file 1 — Supplementary Materials- [file 41392_2023_1531_MOESM1_ESM.docx]

Supplementary Materials for

# Sebaceous gland reprogramming with a single gene, *PPARG*, and small molecules

Yiqiong Liu^1*^, Shuaifei Ji^1*^, Huanhuan Gao^1*^, Huating Chen^1^, Jiangbing Xiang^1^, Shaoyuan Cui^2^, Christos C. Zouboulis^3^, Aizhen Cai^4#^, Xiaobing Fu^1#^, Xiaoyan Sun^1#^

^1^ Research Center for Tissue Repair and Regeneration affiliated to the Medical Innovation Research Department and 4th Medical Center, PLA General Hospital and PLA Medical College; PLA Key Laboratory of Tissue Repair and Regenerative Medicine and Beijing Key Research Laboratory of Skin Injury, Repair and Regeneration; Research Unit of Trauma Care, Tissue Repair and Regeneration, Chinese Academy of Medical Sciences, 2019RU051, Beijing 100048, P. R. China

^2^ Department of Nephrology, The First Medical Center, Chinese PLA General Hospital, State Key Laboratory of Kidney Diseases, Beijing 100048, China.

^3^ Departments of Dermatology, Venereology, Allergology, and Immunology, Staedtisches Klinikum Dessau, Brandenburg Medical School Theodor Fontane and Faculty of Health Sciences Brandenburg, Dessau, Germany

^4^ Institute of General Surgery, Department of General Surgery, the 1st Medical Center, PLA General Hospital, 28 Fu Xing Road, Beijing 100853, P. R. China

**Running title:** PPARG-mediated sebaceous gland reprogramming

^#^**Correspondence:** Xiaoyan Sun, Research Center for Wound Healing & Regenerative Medicine, Department of Medical Innovation, Chinese PLA General Hospital, 28 Fu Xing Road, Beijing 100853, Tel. 0086-10-66936345; Fax: 0086-10-66936345; e-mail: [yanzisun1979@sina.com](mailto:yanzisun1979@vip.sina.com).

**Correspondence may also be addressed to:** Xiaobing Fu, Research Center for Wound Healing & Regenerative Medicine, Department of Medical Innovation, Chinese PLA General Hospital, 28 Fu Xing Road, Beijing 100853, Tel. 0086-10-66936345; Fax: 0086-10-66936345; e-mail: [fuxiaobing@vip.sina.com](mailto:fuxiaobing@vip.sina.com).

Aizhen Cai, Institute of General Surgery, Department of General Surgery, the 1st Medical Center, Chinese PLA General Hospital, 28 Fu Xing Road, Beijing 100853, China; e-mail: [caiwei9248@sina.com](mailto:caiwei9248@sina.com).

*These authors contributed equally.

**The file includes:**

Supplementary figure 1. Human epidermal keratinocytes (HEK) cultures do not contain sebaceous gland cells (SGC).

Supplementary figure 2. PPARγ overexpression directed HEK toward SG fate.

Supplementary figure 3. PPARγ overexpression induced conversion of NHEK into SG-like cells.

Supplementary figure 4. NHEK showed higher conversion efficiency and stemness in compared to HEK.

Supplementary figure 5. 6C treatment elevated the stemness of HEK.

Supplementary figure 6. Both iSC and differentiated iSC possessed normal karyotypes.

**Other Supplementary Material for this manuscript includes the following:**

1. Materials and Methods
2. Table S1. Primer sequences used in the study

**Supplementary figure 1**

**
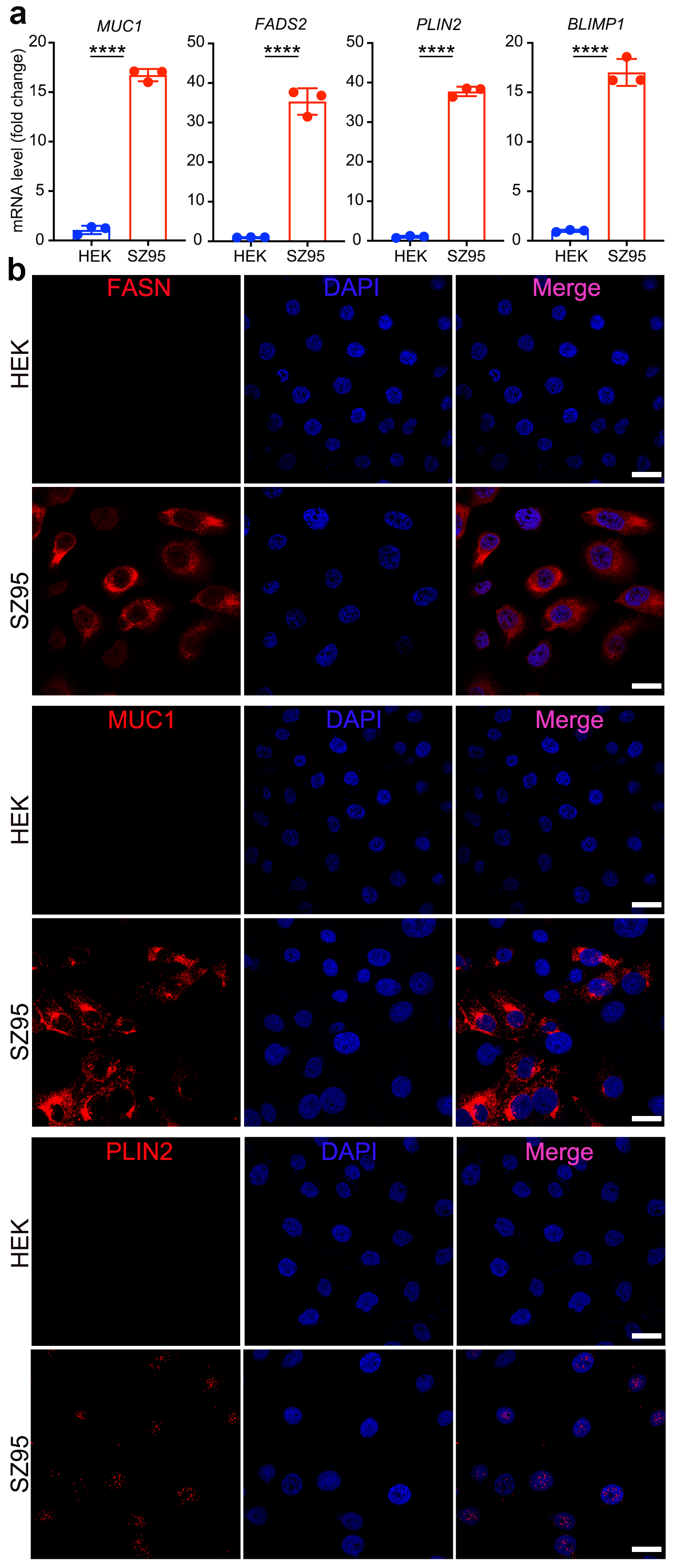
**

**Supplementary figure 1. Human epidermal keratinocytes (HEK) cultures do not contain sebaceous gland cells (SGC).**

**1a.** qRT-PCR analysis of transcriptional expression of the SG-associated genes *MUC1*, *FADS2*, *PLIN2* and *BLIMP1* in HEK and SZ95 sebocytes. Data are expressed as mean ± S.D.; n = 3. ****, *p* < 0.0001; **1b.** Representative immunofluorescence of FASN, MUC1 and PLIN2 in HEK and SZ95 sebocytes. Scale bars = 25 μm.

**Supplementary figure 2**


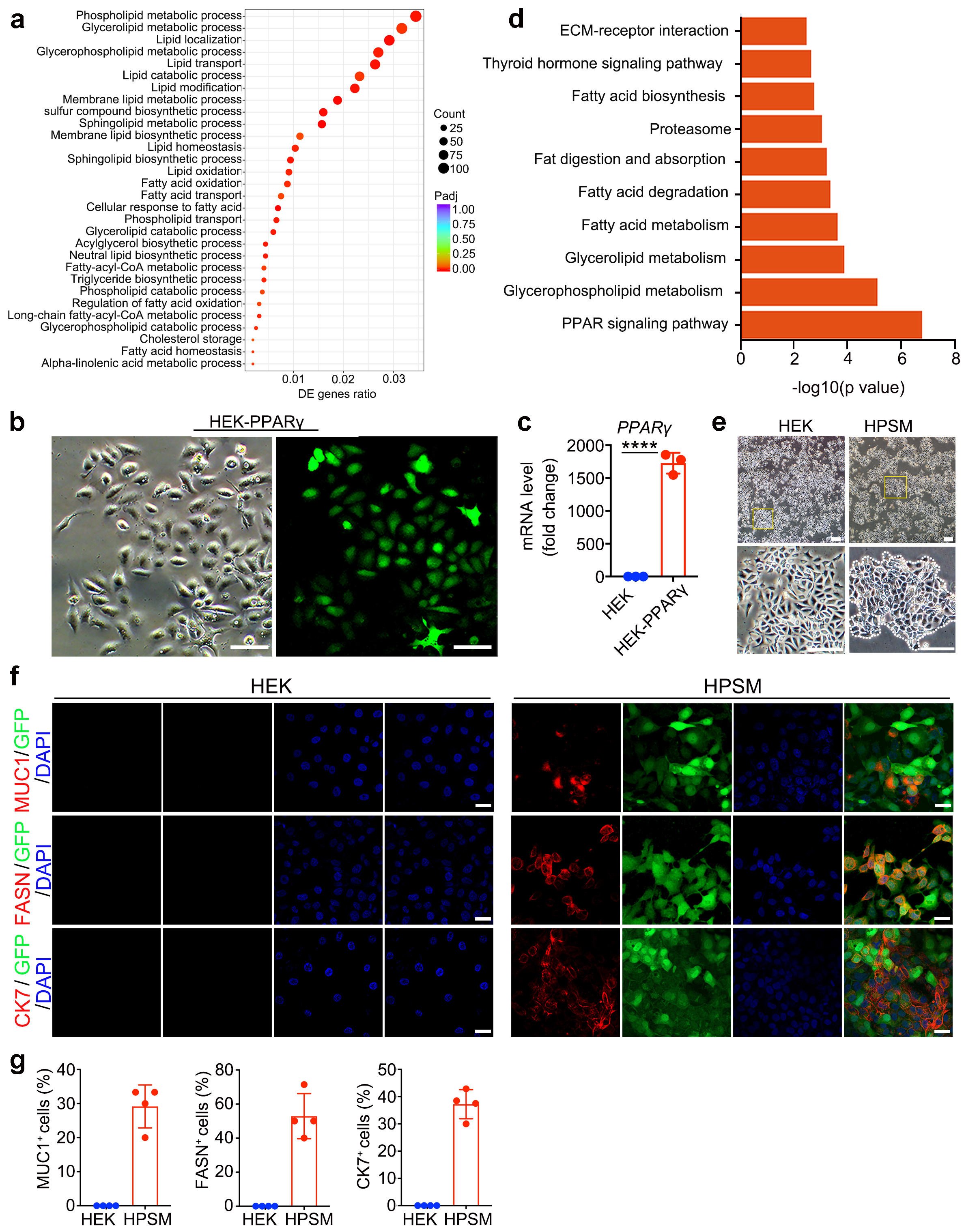


**Supplementary figure 2. PPARγ overexpression directed HEK toward SG fate.**

**2a.** Gene ontology (GO) enrichment analysis of the upregulated differentially expressed genes (DEG) between human primary sebaceous gland cells (pSC) and HEK; **2b.** Phase contrast and fluorescent images of HEK stably overexpressing PPARγ (HEK-PPARγ). Scale bars = 50 μm; **2c.** qRT-PCR analysis of *PPARγ* level in HEK and HEK-PPARγ. Data are expressed as mean ± S.D.; n = 3. ****, *p* < 0.0001; **2d.** KEGG analysis of upregulated DEG in HEK-PPARγ vs. HEK; **2e.** Phase contrast images of HPSM and HEK. Scale bar = 100 μm. Insets, higher magnification of the boxed areas. HPSM, HEK stably overexpressing PPARγ and cultured in SG induction medium (SGIM); **2f.** Representative immunofluorescence of MUC1, FASN, and CK7 in HEK and HPSM. Scale bar = 25 μm; **2g.** Quantitative analysis of MUC1^+^, FASN^+^, and CK7^+^ cells in HEK and HPSM. Quantification was done with four randomly selected individual microscopy fields. Data are expressed as mean ± S.D..

**Supplementary figure 3**

**
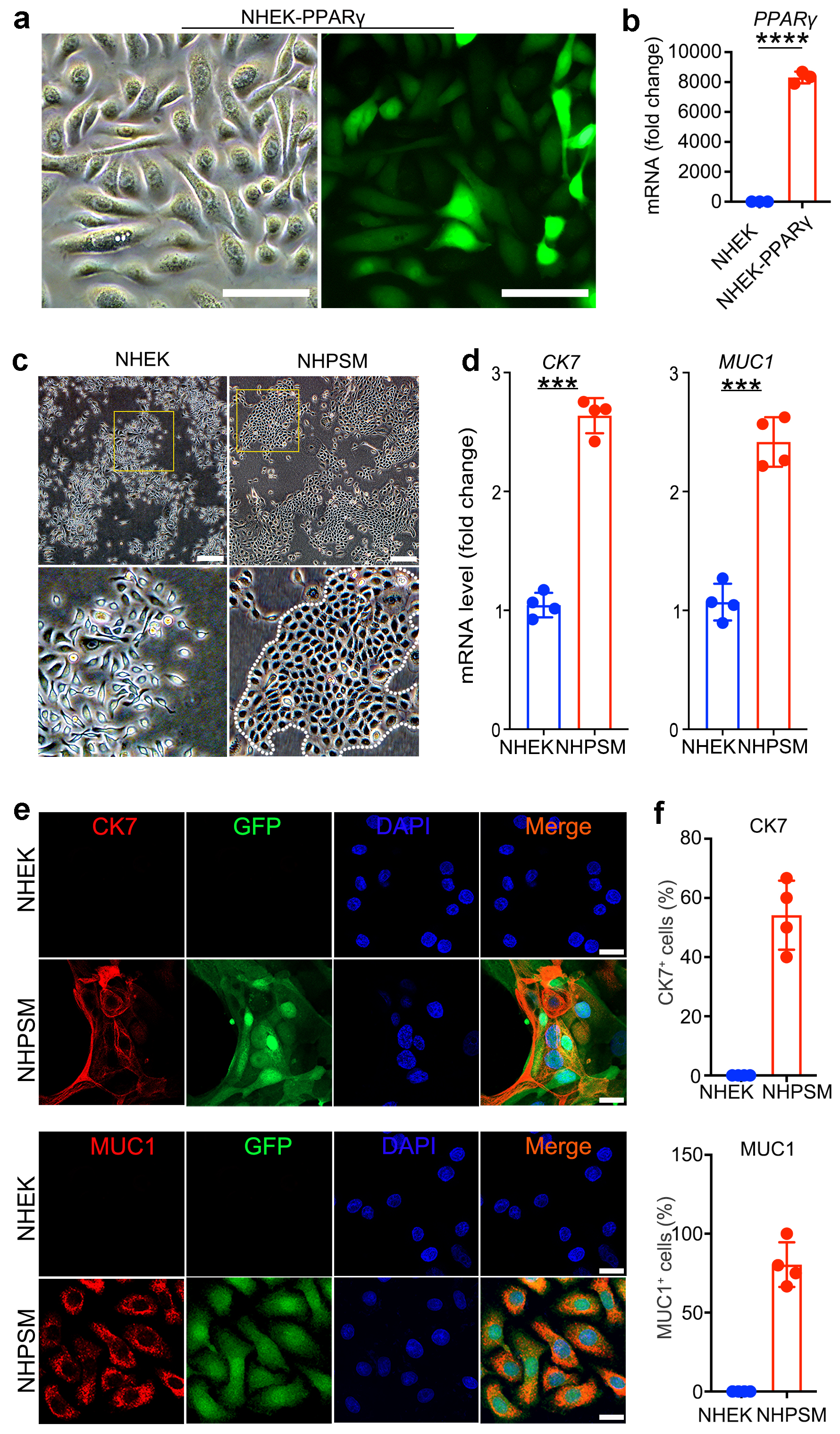
**

**Supplementary figure 3. PPARγ overexpression induced conversion of NHEK into SG-like cells.**

**3a.** Phase contrast and fluorescent images of NHEK stably overexpressing PPARγ (NHEK-PPARγ). Scale bars = 50 μm; **3b.** qRT-PCR analysis of *PPARγ* level in NHEK and NHEK-PPARγ. Data are expressed as mean ± S.D.; n = 3. ****, *p* < 0.0001. NHEK, neonatal HEK; **3c.** Phase contrast images showing the obviously morphological changes of NHPSM compared to NHEK. NHPSM, neonatal HEK stably overexpressing PPARγ and cultured in SGIM. Scale bar = 100 μm. Insets, higher magnification of the boxed areas; **3d.** qRT-PCR analysis of transcriptional expression of *CK7* and *MUC1* in NHEK and NHPSM. Results are expressed as mean ± S.D.; n = 4. ***, *p* < 0.001; **3e.** Representative immunofluorescence of CK7 and MUC1 in NHEK and NHPSM. Scale bar = 20 μm; **3f.** Quantitative analysis of CK7^+^ and MUC1^+^ cells in NHEK and NHPSM. Quantification was done with four randomly selected individual microscopy fields. Data are expressed as means ± S.D..

**Supplementary figure 4**

**
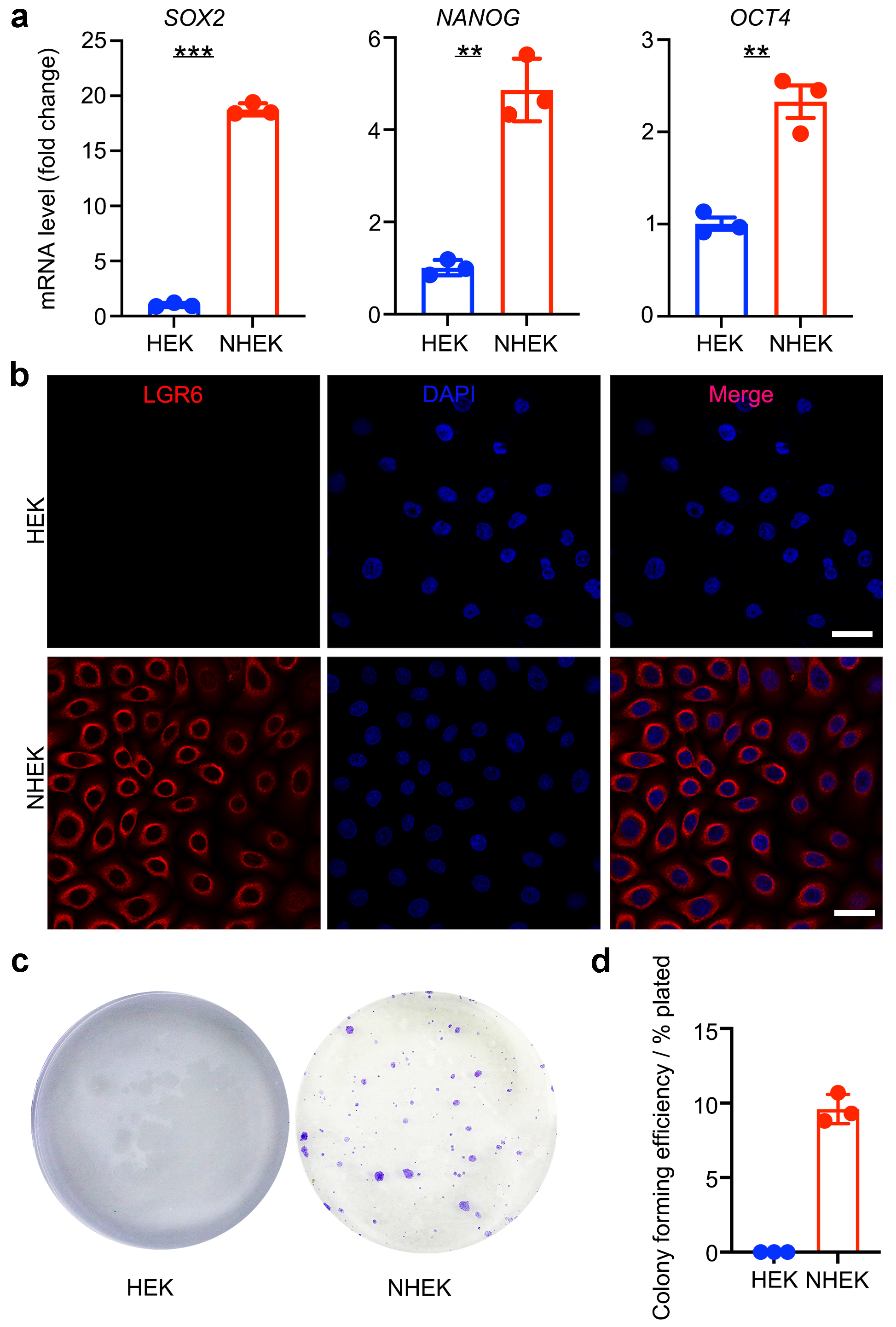
**

**Supplementary figure 4.** **NHEK showed higher conversion efficiency and stemness in compared to HEK.**

**4a.** qRT-PCR analysis of stemness-associated genes *SOX2*, *NANOG*, and *OCT4* in HEK and NHEK. Data are expressed as mean ± S.D.; n = 3. **, *p* < 0.01; ***, *p* < 0.001; **4b.** Representative immunofluorescence of LGR6 in HEK and NHEK. Scale bar = 25 μm; **4c.** Representative images of colony forming unit (CFU) assays in HEK and NHEK. n = 3; **4d.** Quantification of colony forming efficiency in HEK and NHEK. Results are expressed as mean ± S.D., n = 3.

**Supplementary figure 5**

**
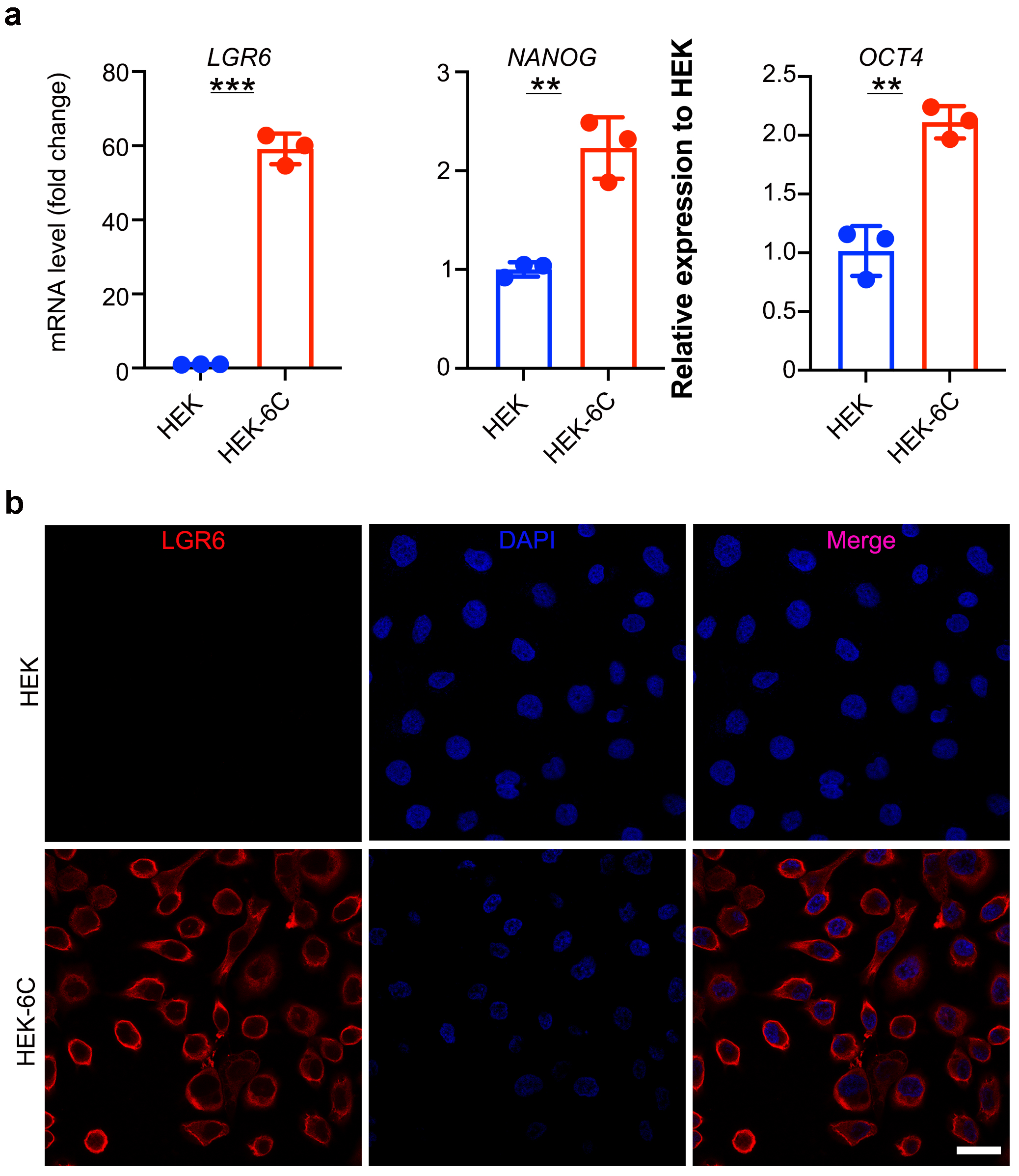
**

**Supplementary figure 5. 6C treatment elevated the stemness of HEK.**

**5a.** qRT-PCR analysis of stemness-associated genes *LGR6*, *NANOG*, and *OCT4* in HEK and HEK-6C. HEK-6C, HEK treated with chemicals 616452 (6) and CHIR99021 (C). Data are expressed as mean ± S.D.; n = 3. **, *p* < 0.01; ***, *p* < 0.001; **5b.** Representative immunofluorescence of LGR6 in HEK and HEK-6C. Scale bar = 25 μm.

**Supplementary figure 6**

**
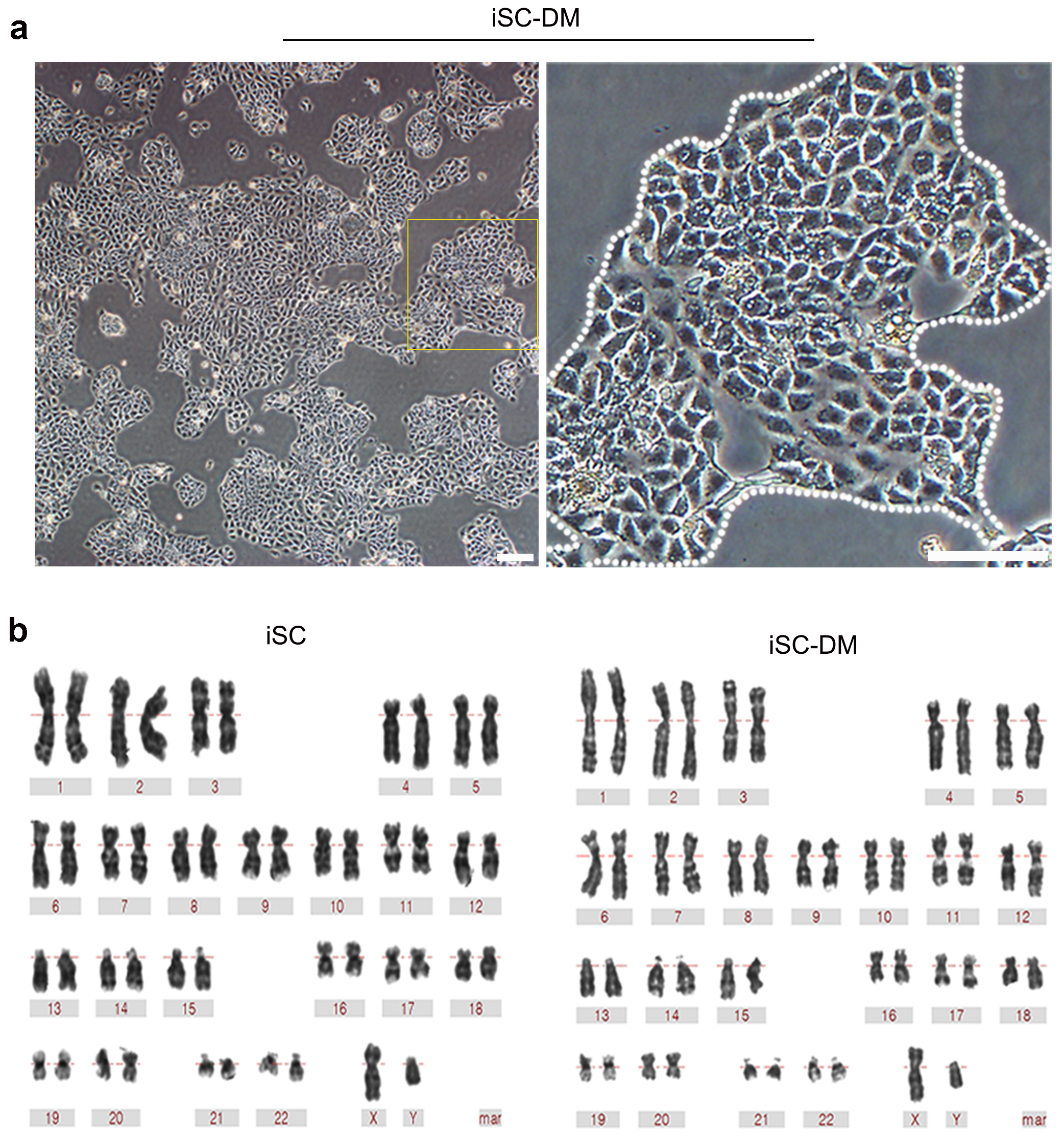
**

**Supplementary figure 6. Both iSC and differentiated iSC possessed normal somatic karyotypes.**

**6a.** Phase contrast images of iSC-DM. Scale bar = 100 μm. Insets, higher magnification of the boxed areas. iSC-DM, iSC cultured in sebaceous gland differentiation medium (DM); **6b.** Karyotype analysis of iSC and iSC-DM in cultures.

# MATERIALS and METHODS

# Cell Culture and Treatment

Primary HEK were isolated from foreskin specimens in males aged 15-20 years old. Briefly, skin tissues were washed with phosphate-buffered saline (PBS) (Solarbio, China) containing 2% penicillin/streptomycin (Solarbio, China), and then cut into pieces and incubated with 0.5% dispase II (Sigma-Aldrich, USA) at 4 °C for 20 h. Separating the epidermis from underlying dermal connective tissue, and digesting the epidermis with 0.25% trypsin for 20 min at 37 °C. After halting the digestion and washing three times with PBS, the single epidermal keratinocytes were resuspended and seeded on T25 culture flasks coated by collagen IV (Sigma-Aldrich, USA) and cultured with growth medium comprising of epilife medium (Thermo Fisher Scientific, USA) supplemented with 1% human keratinocyte growth supplement (Thermo Fisher Scientific, USA) and 1% penicillin/streptomycin (Solarbio, China). Neonatal HEK, from foreskin of newborns, were isolated and cultured as above.

Primary sebaceous gland cells (pSC) were isolated using a modified method reported by T. Kealey.^1^ Briefly, subcutaneous fat of normal human skin samples from breast plastic surgery was removed by blunt dissection. Then the remaining epidermis with the dermis tissues were cut into 5 mm wide pieces. Next, the pieces were cut into very small pieces using sharp scissors. The free sebaceous glands were picked under a dissecting microscope using micro forceps and digested into single cells with Accutase (Sigma-Aldrich, USA). All the procedures were carried out under sterile conditions.

Human SZ95 sebocytes^2^ were cultured in Sebomed basal medium (Sigma-Aldrich, USA) supplemented with 5 ng/ml recombinant human Epidermal Growth Factor (Gibco, USA), 1 mM CaCl_2_ (Gibco, USA) and 1% penicillin/streptomycin (Solarbio, China).

The research in this study conformed to the ethical protocols, and the present study was approved by the Clinical Research Ethics Committee of the General Hospital of PLA (Beijing, China).

# Generation of iSC

A lentiviral expression plasmid encoding *PPARγ* (sequence information: Human, NM_015869) together with packaging plasmid *psPAX2* and envelope plasmid *pMD2.G* were transfected into 293FT cells to produce the viral particles, in which Lipofectamine™ 2000 (Invitrogen, USA) was used. Untransfected cells were eliminated by continuous 1 μg/ml puromycin selection (Gibco, USA) for indicated days. GFP expression was verified with phase contrast images. To generate induced sebaceous gland cells (iSC), HEK transfected with PPARγ were cultured in sebaceous gland cell induced medium (SGIM) with or without the pretreatment of 5 μM 616452 (Selleck, USA) and 5 μM CHIR99021 (Selleck, USA). The SGIM contains 50% epilife medium (Thermo Fisher Scientific, USA) and 50% human sebocyte basal medium supplemented with 1% penicillin/streptomycin (Solarbio, China), 1% Human Keratinocyte Growth Supplement (Thermo Fisher Scientific, USA), 1% Human Sebocyte Growth Supplement (Cell, USA), 20 ng/ml epidermal growth factor (Thermo Fisher Scientific, USA), 10 ng/ml fibroblast growth factor 7 (Thermo Fisher Scientific, USA), and 10 ng/ml fibroblast growth factor 10 (Thermo Fisher Scientific, USA).

# RNA extraction and quantitative real-time PCR

Total RNA was extracted by using TRIzol (Invitrogen, USA) according to the manufacturer’s instructions. The acquisition of cDNA was achieved using a Prime Script RT reagent kit (TaKaRaBio, Japan). Quantitative real-time PCR was performed using the CFX96 Real-Time System (Biorad, USA) Super Real Premix Plus SYBR Green (Tian Gen Biotechnology, China) according to the manufacturer’s instructions. Quantification of target genes was normalized to the expression of β-ACTIN. The primer information is in Table S1, Supporting Information.

# RNA-Sequencing

Sequencing library construction, Illumina sequencing, and data analysis were carried out by Novogene Bioinformatics (Beijing, China). Total RNA was isolated from HEK, HEK-PPARγ, iSC, iSC-DM, and primary SC by using TRIzol (Invitrogen, USA). Total amounts and integrity of RNA were assessed using the RNA Nano 6000 Assay Kit of the Bioanalyzer 2100 system (Agilent Technologies, USA). After purification, the library was constructed by NEBNext^®^ Ultra™ RNA Library Prep Kit for Illumina^®^. After the qualification of the library, sequencing was performed by the Illumina NovaSeq 6000. For each group, two independent biological replicates were used for sequencing. Differential expression analysis of two groups (two biological replicates per condition) was performed using the DESeq2 R package (1.20.0). Gene Ontology (GO) terms and KEGG pathways enrichment analysis of differentially expressed genes were implemented by the clusterProfiler R package (3.8.1). Genes with adjust *P* value < 0.05 and |FC| ≥ 1 are identified as differentially expressed genes.

# Immunofluorescence

For immunofluorescence staining, cells were fixed in 4% paraformaldehyde (Solarbio, China) at room temperature for 30 min and permeabilized in 0.1% triton-X100 in PBS for 10 min. Tissue samples were fixed in 4% paraformaldehyde for 48  h, dehydrated, paraffin-embedded and 4 μm sections were prepared. Antigen retrieval of tissue sections was carried out in 10 mM citric acid buffer (pH = 6.0) for 15 min. Paraformaldehyde-fixed tissue was rinsed in PBS, and then permeabilized. Cells and tissue sections were blocked with PBS containing 0.1% triton-X100 and 5% normal goat serum (Solarbio, China) at room temperature for 12 h. Then cell and tissue sections were incubated with primary antibodies at 4 °C overnight and secondary antibodies at room temperature for 2 h. The primary antibodies used in this study were rabbit anti-FASN (Abcam, USA, 1:300), rabbit anti-MUC1 (Abcam, USA, 1:1000), rabbit anti-CK7 (Abcam USA, 1:150), rabbit anti-LRIG1 (Invitrogen, USA, 1:100), rabbit anti-LGR6 (Abcam, USA, 1:50), rabbit anti-PLIN2 (Invitrogen, USA, 1:200), rabbit anti-Histone H1.0 (Abcam, USA, 1:200), chicken anti-GFP (Abcam, USA, 1:200). The following secondary antibodies were used: goat anti-chicken IgG H&L (Alexa Fluor 488) (Abcam, USA, 1:200), goat anti-rabbit IgG H&L (Alexa Fluor 594) (Abcam, USA, 1:200). Imaging for immunofluorescence involved using a Leica fluorescence microscope.

**Colony forming assay**

HEK without or with 6C treatment were plated in 6-well cell culture plates (Thermo Fisher Scientific, USA) with 1000 cells per well for 14 days. The cells were observed under a microscope and cell clusters greater than 50 cells were counted as a clone. Cells were stained with crystal violet solution (Solarbio, China) following 4% paraformaldehyde fixation (Solarbio, China). After 30 min, the cell colonies were photographed and counted using the ImageJ software as described in our previous study.^3^

# Electron microscopy

Cells were harvested and fixed with 2.5% glutaraldehyde in PBS at room temperature for > 2 h. Cell samples were first dehydrated by a graded series of ethanol for 15 min at each step and transferred to absolute acetone for 20 min. Then resin was used for sample infiltrating and embedding. Samples were prepared as ultrathin sections and observed with the Hitachi TEM system.

# Sebocyte differentiation and Oil Red O staining

Cells were cultured in sebaceous gland differentiation medium (DM) containing SGIM supplemented with 10 μg/ml insulin (Sigma-Aldrich, USA), 100 μM WY-14643 (Selleck, USA), 1 μM Rosiglitazone (Selleck, USA), 10 nM dihydrotestosterone (Selleck, USA) for 12 days and lipid droplets were determined by Oil Red O staining.

Oil Red staining was performed using the Lipid (Oil Red O) staining kit (Solarbio, China) according to the manufacturer’s instructions.

# Skin greasiness assessment

Skin greasiness was measured using Visioscan VC20 plus (Courage-Khazaka Electronic GmbH, Cologne, Germany) according to the manufacturer’s instructions and in accordance with the recommendations of the European Group for Efficacy Measurements on Cosmetics and Other Topical Products (EEMCO). For each value, the arithmetic mean of three consecutive measurements for each subject was calculated.

# Liquid chromatography (LC)-Mass spectrometry (MS) /MS for lipid analysis

**Lipid Extraction**

Lipids were extracted by the modified Folch extraction method by replacing chloroform with methyl-tert-butyl ether (MTBE). Solvents for lipid extraction (LC/MS grade) including MTBE, methanol, and water were purchased from Sigma-Aldrich, USA. Tissue samples were homogenized before lipid extraction. Phase separation was achieved by adding water and centrifugation for 12000 rpm, 15 min. The upper phases were transferred to new tubes, dried under nitrogen flow, and stored at -80°C for analysis.

**Lipidomic analysis**

Lipidomic analysis using LC-MS/MS was performed as previously described.^4^ CORTECS C18 Column (2.1 × 100 mm, Waters) was used for reversed-phase chromatographic separation. Acetonitrile, isopropanol, and water used as follow were all HPLC quality grade. The mobile phase consisted of two solvent mixes. In mobile phase A, 0.77 g ammonium acetate was dissolved in 400 ml water, followed by the addition of 600 ml acetonitrile. For the preparation of Mobile Phase B, 100 ml acetonitrile was mixed with 900 ml isopropanol. Here are the gradient values: 0 min, 37% B; 1.5 min, 37% B; 4 min, 45% B; 5 min, 52% B; 8 min, 58% B; 11 min, 66% B; 14 min, 70% B; 18 min, 75% B; 20 min, 98% B; 22 min, 98% B; 22.1 min 37% B; and 25 min, 37% B.

Lipid data were collected using UHPLC-Q Exactive Orbitrap MS (Thermo Fisher Scientific, USA). Below are the details of the mass spectrometer parameters: spray voltage, 3.2 kV for positive and 2.8 kV for negative; capillary temperature, 320 °C; aux gas flow rate (arb), 10; mass range (m/z), 240-2000 for positive and 200-2000 for negative; full MS resolution, 70,000; MS/MS resolution, 17,500; topN, 10; NCE, 15/30/45; duty cycle, 1.2 s. The LipidSearch software v4.1.16 (Thermo Fisher Scientific, USA) was used for the lipid identification and analysis. Positive mode search was conducted with +H, +NH4 adducts, and negative mode search was conducted using -H, +CH3COO adducts.

Statistical analysis was performed on identified lipids and data were normalized. Principal Component Analysis (PCA) was performed using R package scatterplot3d and heatmap was drawn by R package pheatmap. Together, the first three principal components represented 92.6% of the variance. The clustering in the heatmap was performed using the K-means clustering algorithm for three centers. Based on the maximal intensity in the averaged profile, clusters were assigned to specific sample groups.

# Animals and transplantation

The animal experiment was approved by the Clinical Ethics Committee of the General Hospital of PLA and within the guidelines of the Institutional Animal Care and Utilization Committee. 8-week-old female athymic BALB/c nude mice (Sibeifu Bioscience, Beijing) were used for wound healing assay. Briefly, two equal-sized 6 mm diameter full-thickness cutaneous wounds were created on the dorsal skin with a biopsy punch. 1 × 10^6^ iSC and 2 × 10^6^ human dermal fibroblasts in 100 μl of growth factor-free Matrigel were injected into the dermis at four sites around the wound and topically apply onto the wound bed (n = 40). Mice in the control group were treated the same way but injected with 2 × 10^6^ human dermal fibroblasts and 100 μl of growth factor-free Matrigel (n = 40). Besides, mice in the vehicle group were treated the same way but injected with PBS and 100 μl of growth factor-free Matrigel (n = 40). On day 0, 3, 7, 10, 14, 16, and 18, each wound was photographed, and the wound area was calculated by Image J. On day 71, the mice were sacrificed and the entire wound tissues were harvested for subsequent experiments.

# Statistical analyses

Two-tailed Student’s t-test was conducted to analyze the differences between the two groups, with *P* value < 0.05 considered statistically significant. All analyses were performed with GraphPad Prism 9.

**Table S1. Primer sequences used in the study**

| **Genes** | **Forward primer** | **Reverse primer** |
| --- | --- | --- |
| β-ACTIN | CATGTACGTTGCTATCCAGGC | CTCCTTAATGTCACGCACGAT |
| AR | TTGTCCATCTTGTCGTCTTCG | ACCAAGTTTCTTCAGCTTCCG |
| CK7 | GAGGTCAAGGCGCAGTATGAG | GGAGGTCGTCCCCATGCT |
| MUC1 | ACCATCCTATGAGCGAGTACCC | GCCACCATTACCTGCAGAAAC |
| BLIMP1 | TACATACCAAAGGGCACACG | TGAAGCTCCCCTCTGGAATA |
| PLIN2 | ATGGCATCCGTTGCAGTTGAT | GATGGTCTTCACACCGTTCTC |
| GATA6 | GTGCCCAGACCACTTGCTAT | CCCTGAGGCTGTAGGTTGTG |
| LRIG1 | GGTGAGCCTGGCCTTATGTGAATA | CACCACCATCCTGCACCTCC |
| SOX9 | AAGATGACCGACGAGCAGGAG | TGTTCTTGCTGGAGCCGTTGA |
| PPAR-γ | GACCCAGAAAGCGATTCCTTCA | CACGGAGCTGATCCCAAAGT |
| FADS2 | CAACGTGGAGCAGTCCTTCT | AGTAGCGGCTTCTCCTGGTA |
| LGR6 | GCCTACCAGTGCTGTCCCTA | GGCTTTGAGTCCTCCATCT |
| SOX2 | GCCGAGTGGAAACTTTTGTCG | GGCAGCGTGTACTTATCCTTCT |
| OCT4 | TATTCAGCCAAACGACCATC | TTGTTGTCAGCTTCCTCCAC |
| NANOG | CCCCAGCCTTTACTCTTCCTA | CCAGGTTGAATTGTTCCAGGTC |

AR, Androgen receptor; CK7, Cytokeratin 7; MUC1, Mucin-1; BLIMP1, B-lymphocyte-induced maturation protein 1; PLIN2, Perilipin 2; GATA6, GATA binding protein 6; LRIG1, Leucine rich repeats and immunoglobulin like domains 1; Sox9, SRY-box transcription factor 9; PPAR-γ, Peroxisome proliferator-activated receptor gamma; FADS2, Fatty acid desaturase 2; LGR6, Leucine rich repeat containing G protein-coupled receptor 6; SOX2, SRY-box transcription factor 2; OCT4, Octamer-binding transcription factor 4; NANOG, Nanog homeobox.

**REFFERENCES**

1 Kealey, T. *et al*. The isolation of human. sebaceous glands and apocrine sweat glands by shearing. *Br. J. Dermatol.* **114**, 181-188, (1986).

2 Zouboulis, C.C. *et al*. Establishment and characterization of an immortalized human sebaceous gland cell line (SZ95). *J. Invest. Dermatol.* **113**, 1011-1020, (1999).

3 Chen, H. *et al.* Biomimetic small exosome with outstanding surgical applications for rapid large-scale wound healing and functional sweat gland restoration. *Nano Today* **45**, 101531, (2022).

4 Tang, H. *et al*. Establishment of local searching methods for orbitrap-based high throughput metabolomics analysis. *Talanta* 156-157,163-171, (2016).
